# Evaluation of the Biostimulant Activity of the Microalgae *Chlorella sorokiniana* and *Scenedesmus* sp. and Their Resistance to the Most Widely Used Agricultural Pesticides

**DOI:** 10.1021/acsomega.5c11935

**Published:** 2026-05-13

**Authors:** Karina Rodríguez-Mora, Fabian Villlalta-Romero, Yariela Nuñez-Salazar, Alex Ossa, Mavis L. Montero

**Affiliations:** † Forest Resources Unit, Engineering Research Institute (INII); University of Costa Rica, San Pedro, San José 2060, Costa Rica; ‡ Center for Research in Materials Sciences and Engineering (CICIMA), 27915 University of Costa Rica, San Pedro, San José 2060, Costa Rica; § School of Chemistry, University of Costa Rica, San José, San Pedro 2060, Costa Rica; ∥ Center for Research in Biotechnology, Costa Rican Technological Institute, Cartago 30109, Costa Rica; ⊥ School of Biology, Costa Rican Technological Institute, Cartago 30109, Costa Rica; # School of Chemistry, Costa Rican Technological Institute, Cartago 30109, Costa Rica; ∇ School of Applied Sciences and Engineering, 28008 EAFIT University, Medellín 050022, Colombia

## Abstract

Microalgae are promising bioinputs for sustainable agriculture. This study is relevant because it was evaluated the biostimulant activity of live microalgae and the pesticide tolerance of *Chlorella sorokiniana* and *Scenedesmus* sp. Biostimulant assays revealed significant improvements in plant growth parameters: *Scenedesmus* sp. increased germination by 157% and root elongation, while *C. sorokiniana* induced the greatest cotyledon thickening. Both species enhanced the chlorophyll content at all doses. Pesticide tolerance varied by species and compound: herbicides caused moderate stress, whereas fungicides (especially Mancozeb) caused severe morphological damage. AFM and SEM confirmed membrane alterations under chemical exposure. Overall, these microalgae demonstrated strong biostimulant potential and tolerance to agrochemicals, highlighting their role as tools for crop productivity.

## Graphical abstract

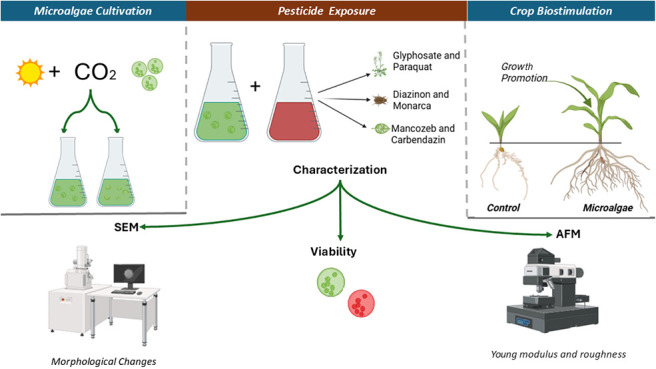

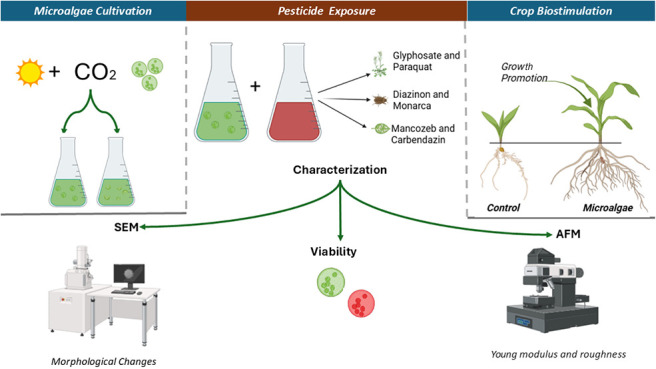

## Introduction

The rapid growth of the global population is driving an unprecedented demand for food, water, energy, and other resources.^1,2^ To meet the rising food requirements, intensive agricultural practices are increasingly relying on synthetic pesticides.^1^ While effective against pests, these compounds are applied at high rates, lack selectivity, and pose significant risks due to their persistence and toxicity.^3^ Excessive pesticide use contributes to nutrient leaching, eutrophication, soil and air pollution, and adverse effects on human health.^4^

Many herbicides induce oxidative stress, which leads to lipid peroxidation, thereby compromising membranes. Even insecticides, though not intended to target photosynthetic processes, have been shown to impair photosynthesis.^5^

This trend is largely driven by an agro-industrial economy and the tropical climate, which requires more frequent applications compared to temperate regions.^6,7^ The consequences are immense, with widespread environmental impacts, economic costs, and a high incidence of poisoning, much of which remains underreported^8^ Developing sustainable agricultural alternatives has therefore become a national priority.

Biological inputs have gained attention as a promising strategy to enhance crop productivity while reducing environmental and health risks.^4,9^ Among them, microalgae are particularly attractive due to their ability to produce phytohormones, bioactive metabolites, and macro- and micronutrients that stimulate plant growth and suppress pathogens.^4,10^

Living organisms applied as monocultures or consortia offer important advantages, as they multiply directly in the soil, require minimal processing, and enhance plant growth parameters. They also fix atmospheric nitrogen and secrete phytohormones that promote growth, suppress pests, and reduce pollutants by mechanisms such as metal immobilization and the degradation of pharmaceutical residues. These effects, however, vary depending on the strain.^11^

Microalgae are particularly relevant due to their production of bioactive compounds with the potential to mitigate environmental impacts and support sustainable agricultural systems.^12^ For this reason, they have been widely proposed as alternatives to chemical fertilizers.^13^ Among the compounds of greatest agronomic interest are phytohormones such as cytokinins (e.g., isopentenyl adenine), gibberellins, and auxins (indole-3-acetic acid), which stimulate root initiation and elongation.^14^ To assess these effects, four biostimulant activity protocols were performed using live microalgae at three different doses.

*Chlorella sorokiniana* is a cosmopolitan freshwater green algae with spherical cells and broad environmental adaptability,^15^ whereas *Scenedesmus* sp. is a colonial species characterized by ellipsoidal or ovoid cells arranged in parallel rows.^16–18^ These microalgae were selected for their potential as agricultural bioinputs.^19^

The objective of this study is to assess different microalgal doses for their capacity to promote plant growth and to identify the dose that produces the optimal biostimulant effect. Subsequently, the influence of agrochemicals on cellular morphology and viability is examined by using the previous dose, with the goal of enabling the integration of live microalgae with agrochemicals to support the transition toward more sustainable farming practices.

## Materials and Methods

### Chemicals

Urea (CAS 57–13–6), potassium nitrate (CAS 7757–79–1), and potassium dihydrogen phosphate (CAS 7778–77–0) for microalgae culture were purchased DISAGRO as agricultural fertilizer grade. For the biostimulant, the reagents Ca­(NO_3_)_2_ (CAS 13477–34–4), KNO_3_ (CAS 7757–79–1), KH_2_PO_4_ (CAS 7778–77–0), Mg_2_SO_4_·7 H_2_O (CAS 10034–99–8), and fluorescein diacetate (FDA) (CAS 596–09–8) were purchased from Sigma-Aldrich. The phytochemical controls, such as gibberellic acid (CAS 77–06–5), indol-3-butyric acid (CAS 133–34–4), and kinetin (6-furfurylaminopurine) (CAS 525–79–1), were purchased from PhytoTech LABORATORIES. High-purity Milli-Q water (18.2 MΩ·cm) was obtained from a Millipore Milli-Q purification system. All solutions were freshly prepared for immediate use in all experiments.

This section describes the detailed procedural steps carried out during and after the experimental assays. The workflow is illustrated in the flowchart provided in Figure S1 of the Supporting Information, which outlines the sequence of operations and the methodologies applied for data processing.

### Strains and Growth Conditions

The selected strains were from the culture collection of the Research Center of Biotechnology of the Costa Rica Institute of Technology, and the selected strains were: *Scenedesmus* sp. (MT35) and *Chlorella sorokiniana* (MT05). Culture of the strains was carried out under environmental conditions in Cartago, Costa Rica, in a small 1 m^3^ tank with continuous air bubbling. The culturing medium was a mixture of urea (0.63g/L), potassium nitrate (0.085g/L), and potassium dihydrogen phosphate (0.192g/L). In the exponential growth phase, 15 days after the culture start, culture density was 6 × 10^6^ cel/mL, and cell density was quantified using a hemocytometer. The biomass was harvested by centrifugation (Thermo Scientific, Centrifuge Sorvall Legend X1R, USA) at 710 × g at 4 °C for 10 min, before being recovered and washed with sterile distilled water.

### Elemental Characterization and X-ray Fluorescence (XRF)

The elemental analysis was carried out using a CHNS/O analyzer (FlashSmart Elemental, Thermo Fisher Scientific, Waltham, MA, USA) based on the modified Dumas method. The analysis was done in triplicate. For the XRF technique, a Bruker AXS S8 TIGER Series 2 (Karlsruhe, Germany) sequential wavelength dispersive X-ray fluorescence spectrometer (WDXRF) was used for direct analysis of the samples.^20^

### Biostimulant Activity Protocols

For the bioestimulante assay, four treatments were evaluated: Positive Control for the assay, Dose 1 (0.50 × 10^6^ cells/mL), Dose 2 (1.00 × 10^6^ cells/mL), and Dose 3 (2.00 × 10^6^ cells/mL).

### Thickening of Cotyledons

Commercial seeds of cucumber (*Cucumis sativus* L.) were used to evaluate the stimulant effect of cytokine.^21^ Cucumber seeds were placed in metal trays with 0.7% agar-solidified KNOP nutrient medium that consisted of 0.8 g/L Ca­(NO_3_)_2_, 0.2 g/L KNO_3_, 0.2 g/L KH_2_PO_4_ and 0.2 g/L Mg_2_SO_4_·7 H_2_O. They were incubated at 25 °C in a dark chamber for 5 days. Once this time had passed, 3 mL of the treatment to be evaluated was added. The cotyledons were cut and separated. The weight of 10 cotyledons was measured before they were transferred to Petri dishes. This was done under green light or in darkness. Water was used as a negative control, and kinetin (1 mg/L) as a positive control; four replicas of each were made. The plates were incubated at 25 °C in the dark for 3 days. The cotyledons were dried on paper and weighed again. A weight comparison was made to calculate weight gain. The weight gain of each extract treatment was compared with that of the negative and positive controls.

### Germination Rate

Germination was studied on the commercial seed Hayslip tomato seeds 493188–55. The method used has been described elsewhere^22^ with some modifications, such as the change of seed type; in this case, tomato was used. The experiments were carried out in modified triplicate, and 25 seeds were used. Distilled water was used as a negative control, and gibberellic acid at 5 mg/L was used as a positive control. They were incubated in the dark at 25 °C for 3 days.^22^ *C. sorokiniana* and *Scenedesmus* spp. were evaluated at different concentrations. The hypocotyl length of each shoot was determined using the ImageJ program, allowing calculation of the germination rate for each treatment relative to the controls using eq 1,

1 $$\mathrm{GI}\left(\%\right)=\frac{G\times L}{G_{W}\times L_{W}}\times 100$$

where *G* and *L* are the number of seeds germinated and the hypocotyl length of each treatment, while *G*_W_ and *L*_w_ correspond to the values obtained with Milli -Q water.

### Root Growth

Adventitious root growth was analyzed in stem cuttings. Two cm of sterile perlite was added and moistened with water, and sorghum seeds were placed evenly on the perlite. After 10 days at 25 °C with 65% humidity and fluorescent lighting, and once a uniform size was reached, the stems were cut 3 cm below the cotyledon. Cuttings were placed in vials with 20 mL of each microalgae. Two plants without roots were placed in each vial, with the leaves left out of the vial. Distilled water was used as a negative control, and IBA 3 mg/L was used as a control; 8 replicates of each treatment were made.

The vials were placed again in light at 25 °C and 65% humidity for 4 days. After incubation, the number of main roots of each cutting, larger than 1 mm, was counted, and each treatment was compared with the controls.^22^

### Effect of Cytokinins on Chlorophyll Content in Corn Leaves

Corn seeds were placed on moistened perlite and incubated at 25 °C with light for 10 days. A 1 cm segment was cut from each plant, starting 3 cm from the apical end. Ten segments were weighed and added to each vial. Distilled water was used as a negative control, and kinetin at 1 mg/L was used as a positive control. The vials were left for 4 days at 25 °C, 65% humidity, and darkness. The corn leaf segments were then removed and dried. The segments were extracted with 8 mL of 95% ethanol for 10 min in a double boiler. They were removed as soon as the ethanol boiled, allowed to cool for a few seconds, and then reheated until the leaves lost their green color. They were allowed to cool, transferred, and gauged with ethanol up to 10 mL. Absorbance at 649 and 669 nm was measured and used in eqs 2 and 3. The following equation was used to perform the calculation.^23,24^

2 $$Ch\;a=13.36A_{664}-5.19A_{649}$$

3 $$Ch\;b=27.43A_{649}-8.12A_{664}$$

### Viability of Microalgae with Pesticides

Different pesticide concentration conditions were evaluated using the same concentration of microalgae. The pesticide concentrations selected for this study were half, once, and twice the field-recommended dose. They were evaluated with 100 × 10^6^ (cells/mL) in each of the tests.

These samples were measured at 72 h. To do this, the modified method of^25^ a 100 μL microalgae sample was taken for each of the different concentrations of pesticides. Ten μL of Fluorescein diacetate (FDA, 10 mg/L in acetone) was added, and the viability was analyzed after about 15 min using a fluorescence microscope. A culture of healthy microalgae was used as a positive control, and microalgae with a dose of a chemical agent that causes their mortality were used as a negative control (Table S1). In Table S2 the description shows the active compound and action mode that each pesticide has.

### Microscopic Characterization of Microalgae

For microscopy sample preparation, the cultures were centrifuged at 3000 rpm for 3 min, after which the supernatant was discarded. The pellet was subsequently washed three times with distilled water to remove the residual pesticides.

### Scanning Electron Microscopy (SEM)

Scanning electron microscopy^26^ was used to determine morphological changes in microalgae. A Zeiss Sigma 300 scanning electron microscope was used, which belongs to the Center for Research in Microscopic Structures (CIEMic). For sample preparation, 20 μL of the previously washed suspension was deposited onto the carbon tape, allowed to air-dry, and subsequently coated with a thin layer of gold.

### Determination of Young’s Modulus

For the quantification of Young’s modulus of *Senedesmus* sp. and *C sorokinana*, UTEX 2505, a Park Systems atomic force microscope, Model XE7, with a B50-NCHR cantilever and an applied force of 28 N/m was used.^27^ A 100-μL aliquot of the sample was placed on a glass microscope slide and allowed to dry in a desiccator.

### Determination of Zeta Potential (ζ)

The zeta potential (ζ) of the aqueous microalgae was determined using an electrokinetic potential analyzer (Zetasizer, Malvern Panalytical). The samples were prepared as a dose of work and homogenized prior to analysis to ensure adequate particle dispersion. All measurements were conducted at a controlled temperature of 25 °C. The zeta potential values were reported as mean ± standard deviation, obtained from repeated measurements, in order to assess the electrostatic stability of the microalgae dispersions in distilled water without culturing media

## Results and Discussion

### Elemental Characterization of Microalgae

The composition of the growth medium has a strong influence on the biochemical profile of microalgal biomass and, consequently, the properties of its derived products.^28^ Table 2 presents the elemental analysis of the two species studied. As expected, carbon and oxygen were the predominant elements, consistent with the accumulation of polysaccharides and lipids in microalgal cells^29^

The average C/N ratios were 6.73 for *Chlorella* and 9.78 for *Scenedesmus*. For comparison, biofertilizers typically present C/N ratios of at least 2.8–17.^30^ These values indicate relatively good ratios in the analyzed strains. However, for agricultural bioinputs, chemical composition alone is not a decisive criterion for assessing their effects on plant growth, crop yield, or nutritional value. Unlike conventional fertilizers, which only incorporate mineral nutrients, the impact of bioinputs is largely mediated by indirect effects on soil processes^31^

When applied to soil, microalgae can promote the formation of microaggregates, release phytohormones, and stimulate atmospheric nitrogen fixation.^32,33^ These mechanisms highlight their potential value as bioinputs, even when elemental ratios differ from those of traditional fertilizers. For this reason, microalgae have a limited application in biofertilizers and the potential to be used as biostimulants.

A detailed profile of the elemental composition of the studied microalgae is presented in Table S1. The main macronutrients detected were Ca, K, and S, while the micronutrients Zn, Fe, and Mg were also present, all of which are essential for growth, stored within the cellular structure, and act as protein cofactors^34–36^

Overall, the elemental composition of *Chlorella sorokiniana* and *Scenedesmus* *sp.* does not indicate hazardous levels of heavy metals or pollutants. Instead, the biomass primarily contains elements essential for development and some accumulated from the growth environment. Combined with CHON analysis, these results reinforce the potential of both species as safe and effective agricultural bioinputs.

### Biostimulant Activity

Since environmental conditions were constant across all treatments, the observed differences can be attributed to the activity of microalgal bioactive compounds. Results are expressed as a percentage relative to the negative control (distilled water), and each experiment also includes a positive control—gibberellic acid, kinetin, or indole-3-acetic acid (IBA), depending on the protocol. Figure 1 illustrates the biostimulant activity. All treatments demonstrated a biostimulant response exceeding that of the negative control.

**Figure 1:**
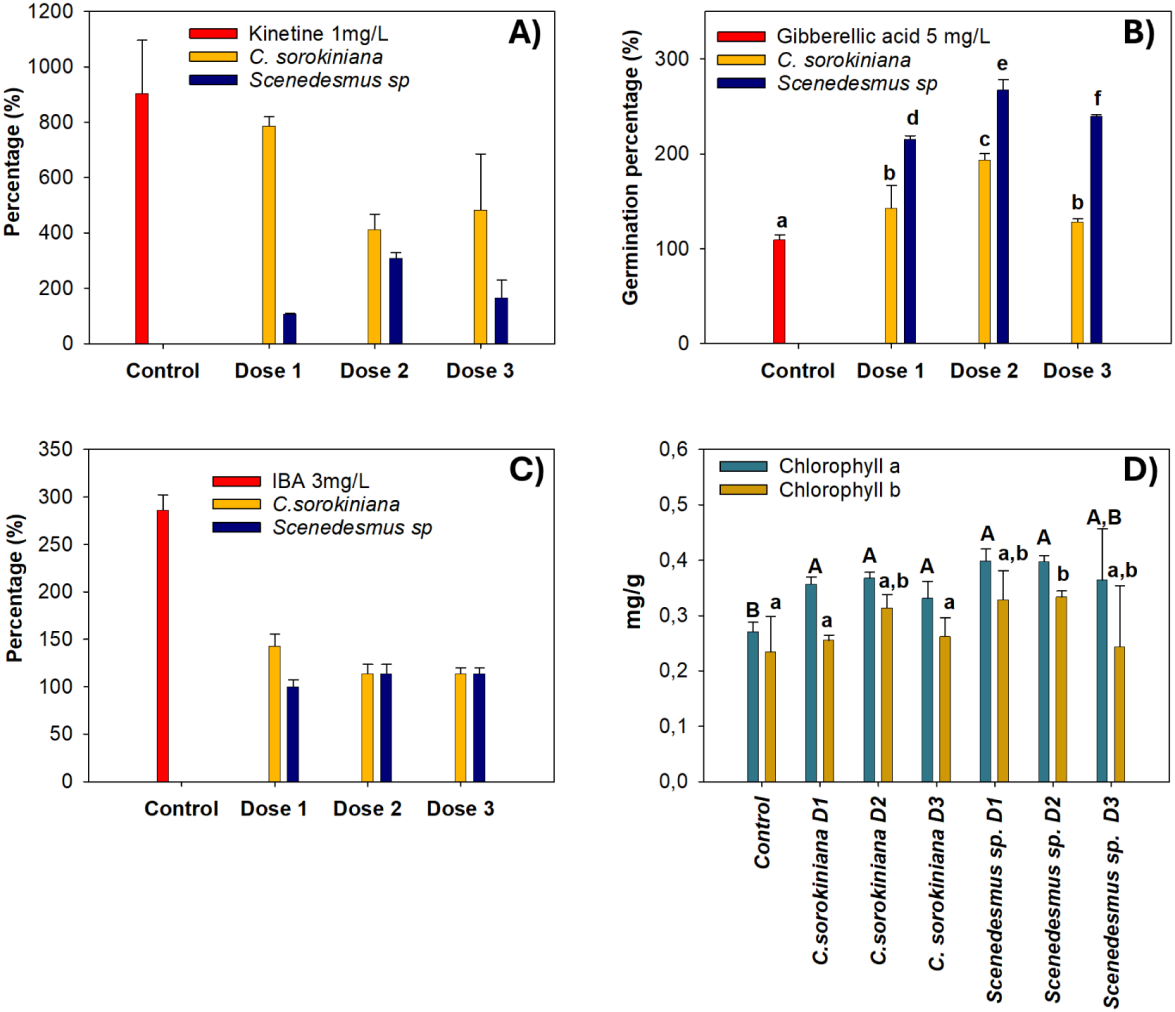
Bioassays: A) Thickening of cotyledons. B) Germination rate. C) Root growth. D) Chlorophyll content. Dose 1 = 0.50 × 10^6^ cells/mL, Dose 2 = 1.00 × 10^6^ cells/mL, and Dose 3= 2.00 × 10^6^ cells/mL. Different letters indicate significant differences (*p* < 0.05) within each treatment.

Figure 1A shows the effect on cotyledon thickening. *C. sorokiniana* induced the strongest impact, with dose 1 performing most efficiently and showing no significant difference from the positive control. In *Scenedesmus* *sp*., dose 2 produced the best response; however, these effects are not sufficient to match the response observed in the positive control. An inhibitory effect was observed at higher concentrations, which is consistent with the fact that microalgae are rich in amino acids, vitamins, minerals, and bioactive metabolites such as phytohormones that, in excess, can reduce rather than enhance stimulation.^37^

The germination index (Figure 1B) confirmed that all doses of both types of microalgae were significantly higher than those of the positive control. The highest responses were obtained at dose 2 (1 × 10^6^ cells/mL), corresponding to increases of 84% for *C. sorokiniana* and 157% for *Scenedesmus* *sp*. Other studies show that microalgal biomass at 0.1 g/mL increased by ∼40%. This suggests that the strains and doses used here are more effective than those reported previously. A reduction at dose 3 again indicated an inhibitory effect of germination at higher concentrations.

For root growth (Figure 1C), all treatments demonstrated biostimulant activity. In *C. sorokiniana* the lowest dose produced the strongest response, whereas in *Scenedesmus* *sp*, Dose 2 was most effective. Both species, however, showed lower values than the positive control. Although both microalgae induced only modest effects on the number of roots, the average root length showed substantial improvement: 1.93 ± 0.26 mm in control compared to 9.2 ± 2.4 mm with *C. sorokiniana* and 12.2 ± 3.6 mm with *Scenedesmus* *sp*. Thus, while fewer roots were generated, they were up to 8–11 times longer than those in the control.

Auxins are well-known to stimulate root initiation and elongation. Vildanova et al. (2023) reported that *Chlorella sorokiniana* increased secondary root formation in tomato and cucumber through positive plant–microalgae interactions.^43^ The same authors also showed that *C. vulgaris* enhanced cucumber root biomass due to phytohormone release, consistent with evidence that members of the genus *Chlorella* secrete compounds that regulate root growth and development.^38^

Regarding chlorophyll content, significant increases in chlorophyll a were observed with both microalgae at all doses relative to the control. For chlorophyll b, the greatest increase occurred with dose 2 of both species, although no significant differences were detected between the two doses. These results are comparable to those of previous studies, where biofertilizers derived from *Chlorella sp.* and *Anabaena* enhanced chlorophyll content in Chinese cabbage (*Brassica rapa* subsp. *pekinensis*), showing increases of 29.2% in chlorophyll a and 33.5% in chlorophyll b compared to unfertilized controls.^39^

### Pesticide Resistance

The viability assay is particularly relevant, as it quantifies the percentage of cells that preserve membrane integrity without rupture or chemical lysis. These results can be directly correlated with the Young’s modulus values obtained through AFM, which reveal morphological changes at the membrane level induced by stress factors such as pH, pollutants, temperature, and salinity. AFM-based mechanical testing thus provides valuable insights into cell wall elasticity and stiffness, allowing for a more precise assessment of structural alterations under chemical stress^40^

This is further illustrated in Figure 2, where error bars denote significant differences between treatments, highlighting the differential tolerance of each microalga. Both microalgae demonstrated good resistance to the two herbicides tested (Figure 2A). However, resistance to Paraquat was lower than glyphosate, consistent with its mechanism of action, because it interferes with the PSI electron transport chain, leading to superoxide anion production, oxidative stress, and subsequent membrane disruption and cell death.^41^ In contrast, *Scenedesmus* *sp.* showed greater tolerance, suggesting species-specific resistance mechanisms.

**Figure 2:**
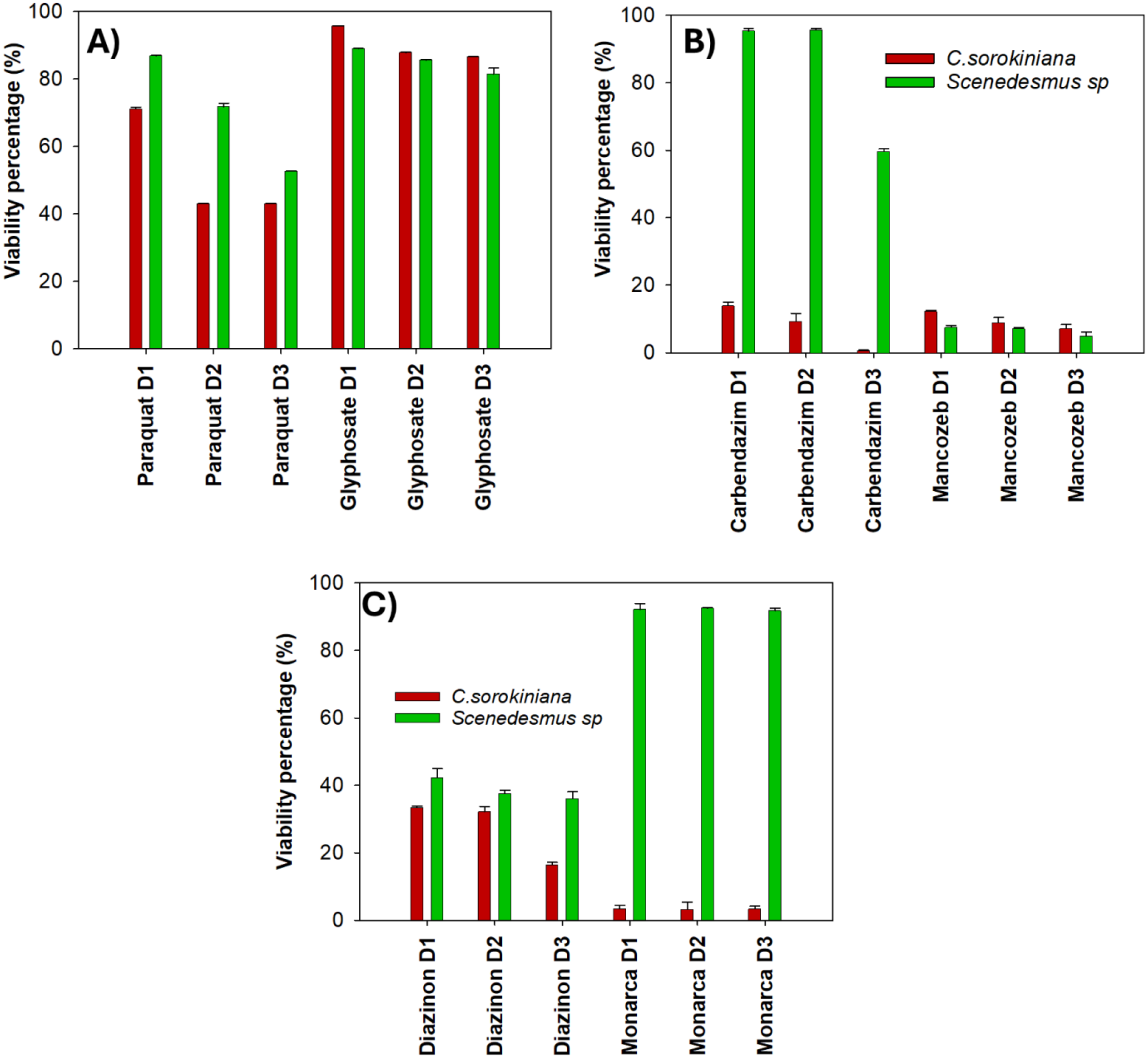
Viability of microalgae using different pesticides and doses: A) Herbicides. B) Fungicides. C) Insecticides. The dose used for each of the pesticides was described in Table 1. All treatments present significant differences (*p* < 0.05) when compared between microalgae exposed to the different pesticides Table 2.

The observed decrease in Young’s modulus for paraquat treatments can be attributed to its direct oxidative damage to membranes, which not only reduces cell viability but also compromises membrane integrity. Glyphosate, despite being a broad-spectrum herbicide, acts by inhibiting the synthesis of essential amino acids via the shikimate pathway, an indirect mechanism. Consequently, glyphosate caused comparatively higher viability and better membrane preservation than paraquat. The description of the mode of action for all pesticides used is in Table S3­(Table 1,).

**Table 1:** Identification of Pesticides and Doses Evaluated with the Two Microalgae

Herbicide			Fungicide			Insecticide		
Identification		Dose (ppm)	Identification		Dose (ppm)	Identification		Dose (ppm)
Paraquat	D1	1000	Carbendazim	D1	375	Diazinon	D1	375
	D2	2000		D2	750		D2	750
	D3	4000		D3	1500		D3	1500
Glyphosate	D1	1000	Mancozeb	D1	1333	Monarca	D1	281
	D2	2000		D2	6666		D2	563
	D3	4000		D3	13333		D3	1126

**Table 2:** Elemental Characterization of Microalgae

	*Chlorella sorokiniana*	*Scenedesmus* *sp*
**C%**	48.14 ± 0.77	43.78 ± 0.45
**H%**	7.03 ± 0.14	6.83 ± 0.27
**O%**	29.60 ± 1.33	31.67 ± 1.24
**N%**	7.16 ± 0.18	4.47 ± 0.07
**C/N**	6.73	9.78

*C. sorokiniana* shows a decrease in the viability to evaluate fungicides (Figure 2B). In contrast, *Scenedesmus* sp. exhibits variable responses depending on the pesticide’s mode of action. Carbendazim exerts its effect at the onset of cell division,^12,32^ accordingly, *C. sorokiniana* shows greater susceptibility to this pesticide due to its higher cell division rate.^11^

Moreover, as observed in the Young’s modulus in Figure 3, *C. sorokiniana* exhibits a reduction in the Young’s modulus, which is related to membrane-level damage; therefore, it not only shows greater susceptibility but also the integrity of the membranes in living cells is also compromised. In contrast, *Scenedesmus* sp. does not significantly differ from the control in this regard. The data on the distribution of the Young’s modulus is in Figure S2 and S3.

**Figure 3:**
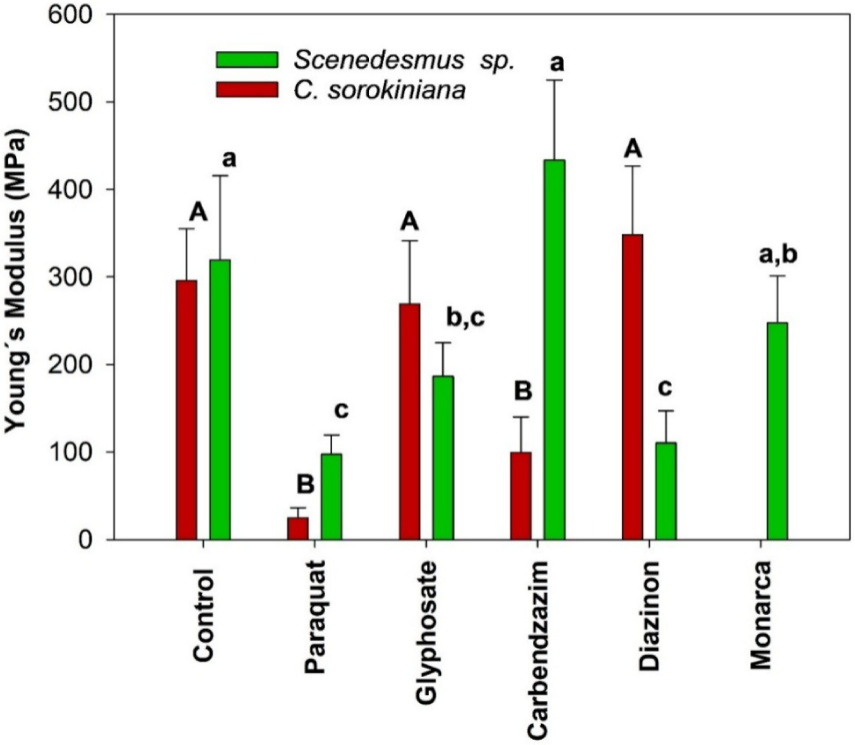
Young’s module *C. sorokiniana* and *Scenedesmus sp.* when applying different pesticides to microalgae.

Although both microalgae are severely impacted by Mancozeb, a nonsystemic fungicide that alters cellular metabolism, it should be noted that the field work doses are quite high, meaning there is very little chance of survival. However, because of the large dosage and insolubility, it is difficult to separate the microalgae from the pesticide, Mancozeb.

For the study with insecticides shown in Figure 2C, it is denoted that *Scenedesmus* *sp.* presents greater resistance, which is also favored by having a more rigid membrane. For diazinon, the involvement in the two microalgae is similar, but *C. sorokiniana* not only shows tolerance, but also demonstrates that it can contribute to treating traces of this pesticide by degrading it into less harmful compounds^42^ For this reason, it may be that when analyzing Young’s modulus, no significant change in the strength of the membrane is observed for *C. sorokiniana*, but in *Scenedesmus* *sp*., it decreases significantly.

For Monarca, the *C. sorokiniana* shows the lowest viability, this could also be reflected in a problem determining Young’s modulus, since it presented such a strong interaction with the membrane. Residue remained, forming a halo despite the washes. For this reason, it was impossible to measure it accurately (see Figure S3).

This interaction may also be due to the microalgae’s zeta potential. *Scenedesmus* has a value of −26.1 ± 4.7, while *Chlorella* has a lower value of −17.9 ± 4.4. Microalgae with a lower zeta potential, like Chlorella, tend to be less stable, more prone to aggregation, and less electrostatically repulsive, making them more susceptible to interaction with the active ingredients of the pesticides benzyfluthrin and thiacloprid. *Scenedesmus* sp. did not show a significant difference.

The roughness of the membranes, as shown in Tables 3 and Table S4 and Figures S4 and S5, is another point to emphasize. In this instance, if the values are analyzed, you could establish a trend of the structural effects on *Chlorella* in the following order: paraquat > carbendazim > glyphosate > diazinon, which would be consistent with the values of Young’s modulus obtained. This would imply that the rougher the surface, the greater the change in Young’s modulus, and consequently, the greater the effect at the membrane level. If we apply the same analysis to *Scenedesmus* sp., we obtain glyphosate > diazinon > paraquat > Monarca > carbendazim. In this case, there are no differences between paraquat, glyphosate, and diazinon at the level of Young’s modulus, but we do notice a trend in terms of roughness because the samples with fewer changes are Monarca and carbendazim; therefore, the same is true as in *C. sorokiniana*. Each treatment was analyzed by scanning microscopy and atomic force microscopy.

**Table 3:** Roughness Values for Microalgae with Differen Treatments in (nm)

Sample		Control	Paraquat	Glyphosate	Carbendazim	Diazinon	Monarca
*C. sorokiniana*	Sa	0.1670	0.5410	0.4080	0.4962	0.3709	-
	Sq	0.2019	0.6260	0.4885	0.5865	0.4293	
	Sq	0.9200	3.3610	2.1246	2.0757	1.5056	
*Scenedesmus* *sp.*	Sa	0.0213	0.1812	0.3558	0.0636	0.2640	0.0971
	Sq	0.0930	0.2078	0.4101	0.0752	0.3319	0.1273
	Sz	0.2494	0.7256	1.4661	0.2850	1.7522	0.6966

Figure 4 shows all morphological changes with the use of pesticides on microalgae. For the herbicides, *C. sorokiniana* has a rougher surface and tends to aggregate in the presence of glyphosate (Figure 4E). This is expected because, as previously indicated, the species is more prone to aggregation when its Z potential is lower. Although *Scenedesmus* sp. exhibits surface modifications, they are not as noticeable.

**Figure 4:**
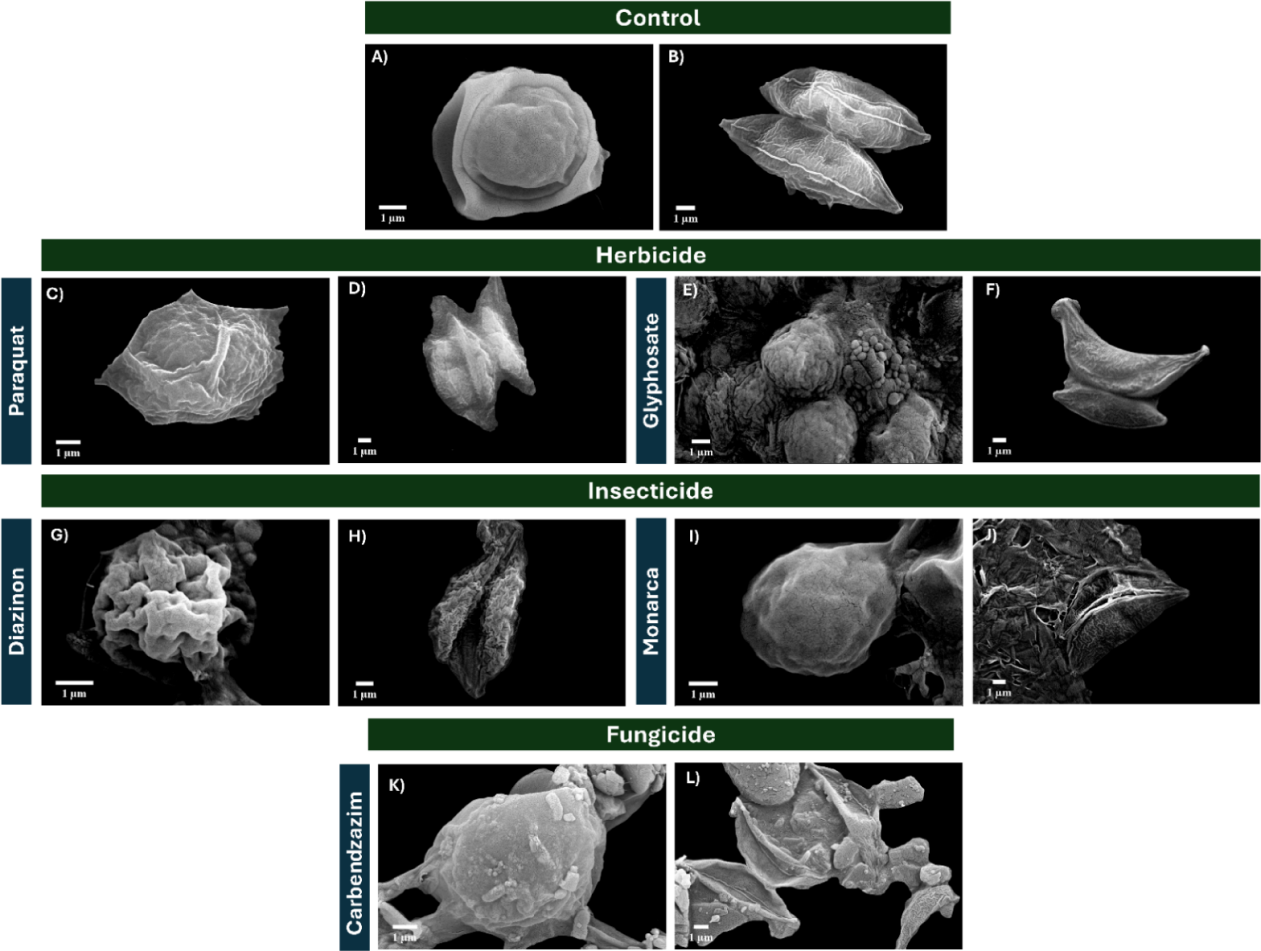
Micrography of electron microscopy for microalgae with pesticides on *C. sorokiniana* and *Scenedesmus* sp.

With the use of the insecticide diazinon, a clear morphological shift was observed, as the cells transitioned from a relatively smooth surface to a highly wrinkled and irregular structure, as shown in Figure 4G and H. Whereas Monarca observed a high interaction; *C. sorokinana* showed something similar to a film on the surface, and in *Scenedesmus* sp., the microstructure displayed disrupted morphology characterized by cracks, folds, and collapsed regions, indicating a loss of integrity. Additionally, Figure 5 shows the morphology observed using the AFM technique, where crystals on microalgae were noted.

**Figure 5:**
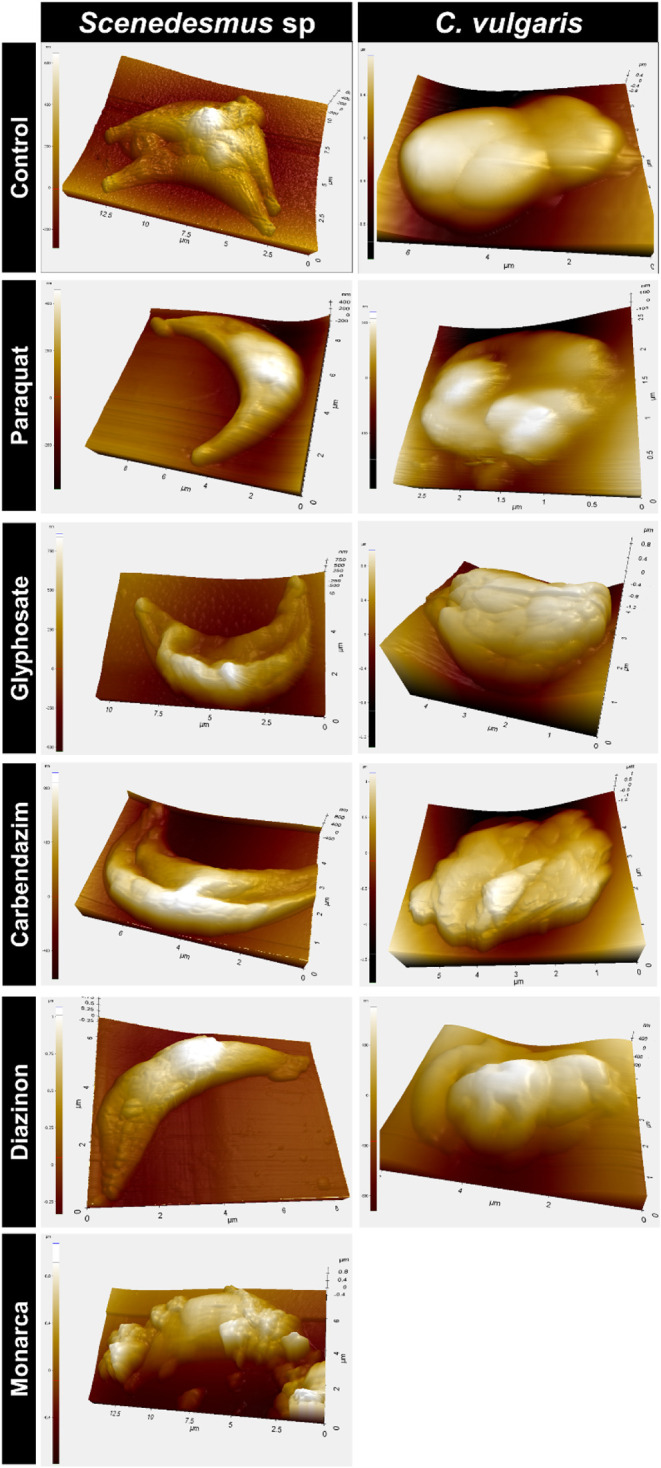
AFM microscopy for determining the morphology of *Scenedesmus* sp. and *C. sorokiniana*.

The experiment with fungicides (Figure 4 K and L) shows that the microalgae have pesticide crystals attached to or close to them. However, *C. Sorokiniana* is the one that most clearly shows deformation and adhesion on its surface from the remaining crystals of Carbenzazin. The Mancozeb treatment could not be observed due to the excess pesticide residue.

## Conclusions

The results demonstrate the biostimulant responses of *Scenedesmus* sp. and *Chlorella sorokiniana* under the experimental conditions evaluated. *Scenedesmus* sp. promoted seed germination and primary root elongation, whereas *C. sorokiniana* primarily induced cotyledon thickening at low application doses. Both microalgae increased the chlorophyll content during early plant development.

When exposed to agrochemicals, both species exhibited differential sensitivity, showing greater tolerance to herbicides compared with fungicides and insecticides. AFM and SEM analyses evidenced surface roughness alterations and localized membrane damage under chemical stress, which were more pronounced in *C. sorokiniana*.

Overall, these findings indicate that microalgae-based bioinputs support their potential use as complementary biostimulants within integrated and sustainable agricultural management strategies.

## Supplementary Material



## Notes

XRF characterization of microalgae, viability values to assess microalgae control, description and identification of pesticides, behavior of microalgae’s population for the determination of Young’s Modulus, halo covering the *C. vulgaris* with pesticide, taken with a Zeiss Axiovert 40 MAT microscope, AFM microscopy for determining the roughness, and analysis of roughness data (PDF)
