# Supplementary material for: Evaluation of the Biostimulant Activity of the Microalgae Chlorella sorokiniana and Scenedesmus sp. and Their Resistance to the Most Widely Used Agricultural Pesticides
Source: ACS Omega. 2026 May 13;11(20):29541–9. doi: 10.1021/acsomega.5c11935 (PMC13216929; doi:10.1021/acsomega.5c11935)
Supplement: Supplementary file 1 [file ao5c11935_si_001.pdf]

Supporting Information

Evaluation of the biostimulant activity of the microalgae Chlorella sorokiniana and Scenedesmus sp and their resistance to the most widely used agricultural pesticides

Karina Rodríguez-Mora[a,b,c], Fabian Villlalta-Romero[d,e,f], Yariela Nuñez-Salazar[d], Alex Ossa[g], Mavis L. Montero[b c]*

a) Forest Resources Unit, Engineering Research Institute (INII); University of Costa Rica; San Pedro, San José, Costa Rica. 2060
b) Center for Research in Materials Sciences and Engineering (CICIMA), University of Costa Rica; San Pedro, San José, Costa Rica. 2060
c) School of Chemistry, University of Costa Rica; San Pedro, San José, Costa Rica. 2060
d) Center for Research in Biotechnology, Costa Rican Technological Institute, Cartago, Costa Rica. 30109
e) School of Biology, Costa Rican Technological Institute, Cartago, Costa Rica. 30109
f) School of Chemistry, Costa Rican Technological Institute, Cartago, Costa Rica. 30109
g) School of Applied Sciences and Engineering, EAFIT University, Medellín, Colombia. 050022

*email: fvillalta@itcr.ac.cr

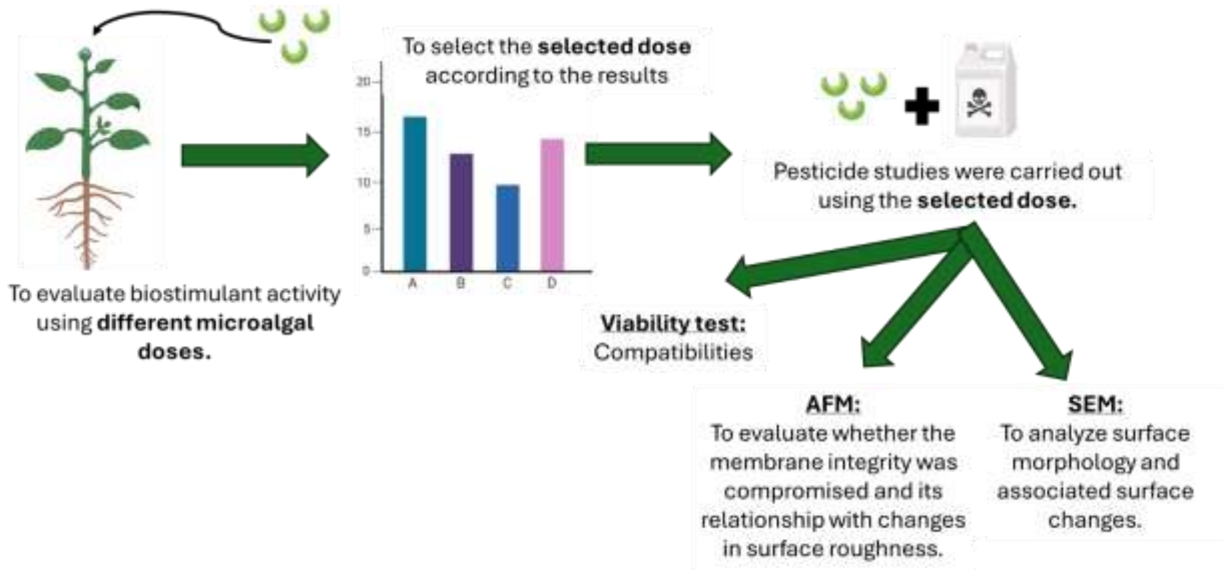

Figure S1. Flowchart the experiment process

Table S1. XRF characterization of microalgae

C. sorokianiana		Scenedesmus sp	
Zn 30.7%	Si 0.54%	Zn --	Si --
Ca 25.4%	Ba 0.25%	Ca 16.8%	Ba --
K 22%	Cl 0.24%	K 44.2%	Cl 3.45%
P 12.4%	Mn 0.19%	P 9.67%	Mn 1.28%
S 5.10%	Mo 0.11%	S 11.4%	Mo --
Fe 1.07%	At 0.11%	Fe 10.1%	At 0.33%
Sr 0.93%	Ti 773ppm	Sr 0.37%	Ti 0.28%
Mg 0.66%	Cu 626ppm	Mg 1.28%	Cu 0.22%

Table S2. Viability values to assess microalgae control.

			C. sorokianana		*Scenedesmus sp*	
Type	Compound	Dose (ppm)	Viability	Standard deviation	Viability	Standard deviation
Control	Azithromycin	0	96.15	0.33	99.00	0.53
		25	84.60	0.19	53.76	0.24
		50	48.49	1.90	32.56	0.90
		75	0.66	0.02	25.48	2.60

Table S3. Description and Identification of pesticides

	Active Component	Mode of action
Herbicide	Paraquat: Dichloride salt of the 1,1'-dimethyl-4,4'bipyridinium ion	It is a non-selective herbicide that acts by contact with the leaves and young green parts of weeds, causing cellular destruction. It diverts the flow of electrons from photosystem 1, generating free radicals and reactive oxygen species. It only exerts action on the tissue contacted; there is no systemic effect:
	Glyphosate-isopropylammonium belongs to the chemical group of organophosphonates	Non-selective broad-spectrum systemic herbicide: any plant can absorb it through its tissues. The chemical agent prevents the affected plant from producing the proteins necessary for its growth, leading to death.
Fungicide	Carbendazim, chemical group of Benzimidazoles	Systemic fungicide, with quick penetration, broad spectrum. It prevents pathogens from dividing normally by interfering with beta tubulin assembly during mitosis
	Mancozeb: Coordination product of zinc ion and manganese ethylene bis dithiocarbamate	It is a contact fungicide that acts preventively, altering cell membrane functions and inhibiting the respiration of fungi
Insecticide	Diazinon: O, O-diethyl O-2-isopropyl-6-methylpyrimidin-4-yl phosphorothioate, belongs to the organophosphorus group	It acts through contact and ingestion, affecting the central and peripheral nervous systems of insects, causing paralysis
	Monarca: Thiacloprid belongs to the Neonicotinoid group, and Beta-Cyfuthrin to the Pyrethroid group.	Mix two active ingredients with systemic and contact action. Neuroactive pesticides

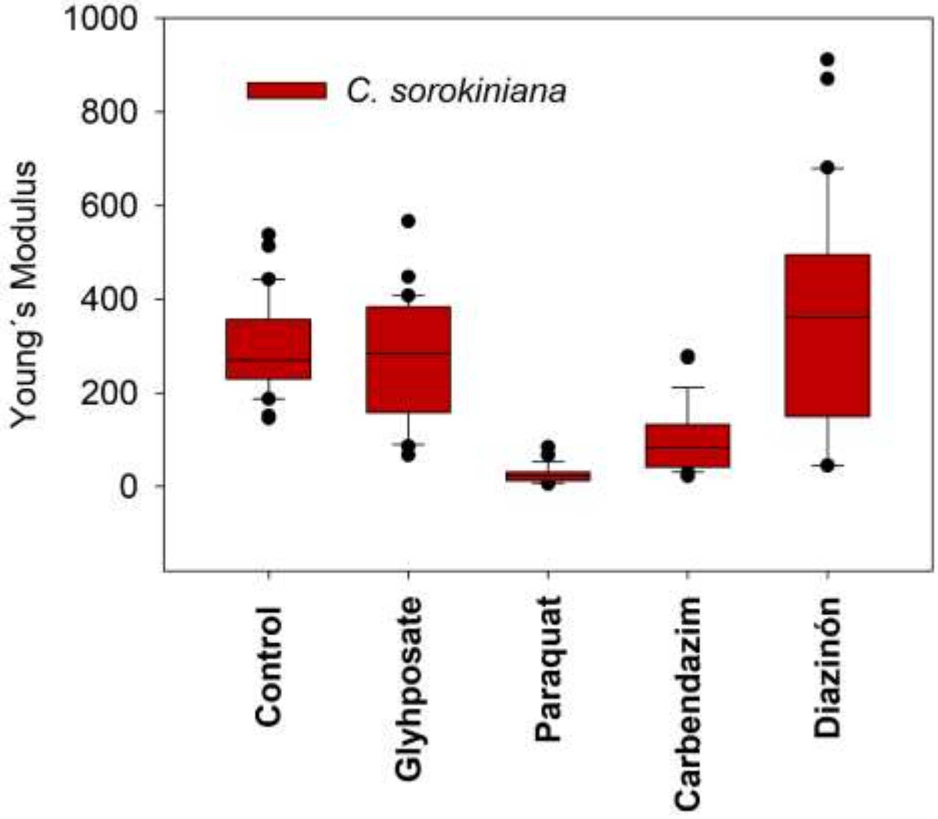

Figure S2. Behavior of the *C. vulgaris* population for the determination of Young's Modulus. Microalgae with water was used as a reference control.

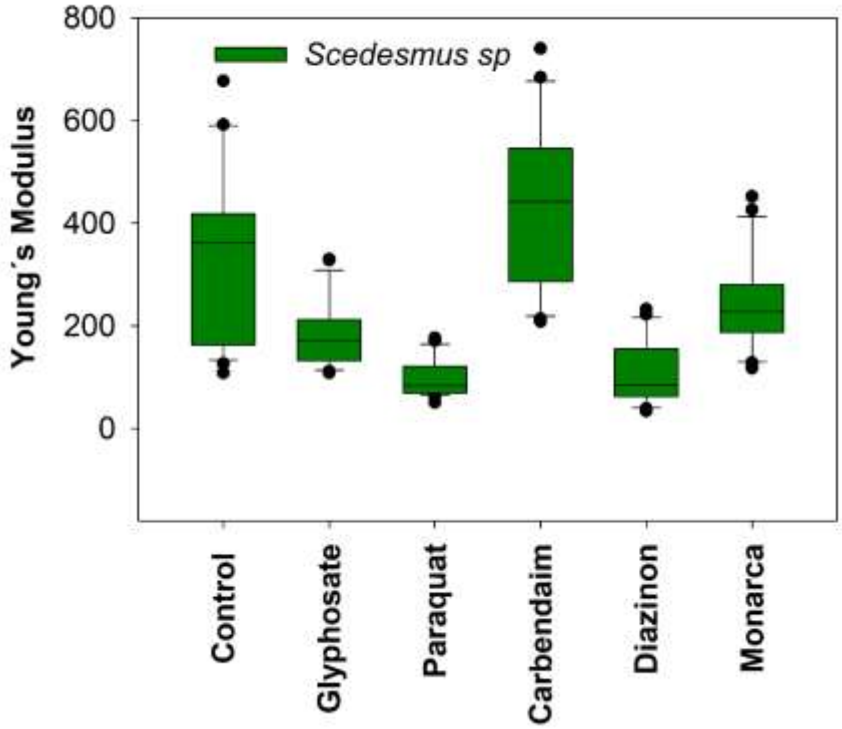

Figure S3. Behavior of the *Scenedesmus sp.* population for the determination of Young's Modulus. Microalgae with water was used as a reference control.

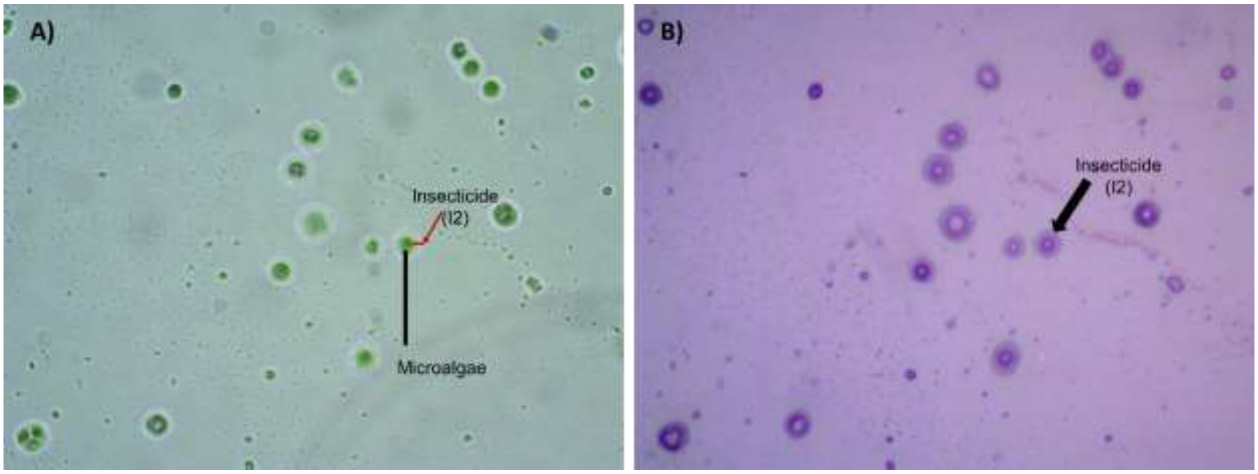

Figure S4. Halo covering the *C. vulgaris* with pesticide, taken with a Zeiss Axiovert 40 MAT microscope. A) Microalga, transmitted light microscopy and halo B) halo surrounding the microalgae, by reflected ligh

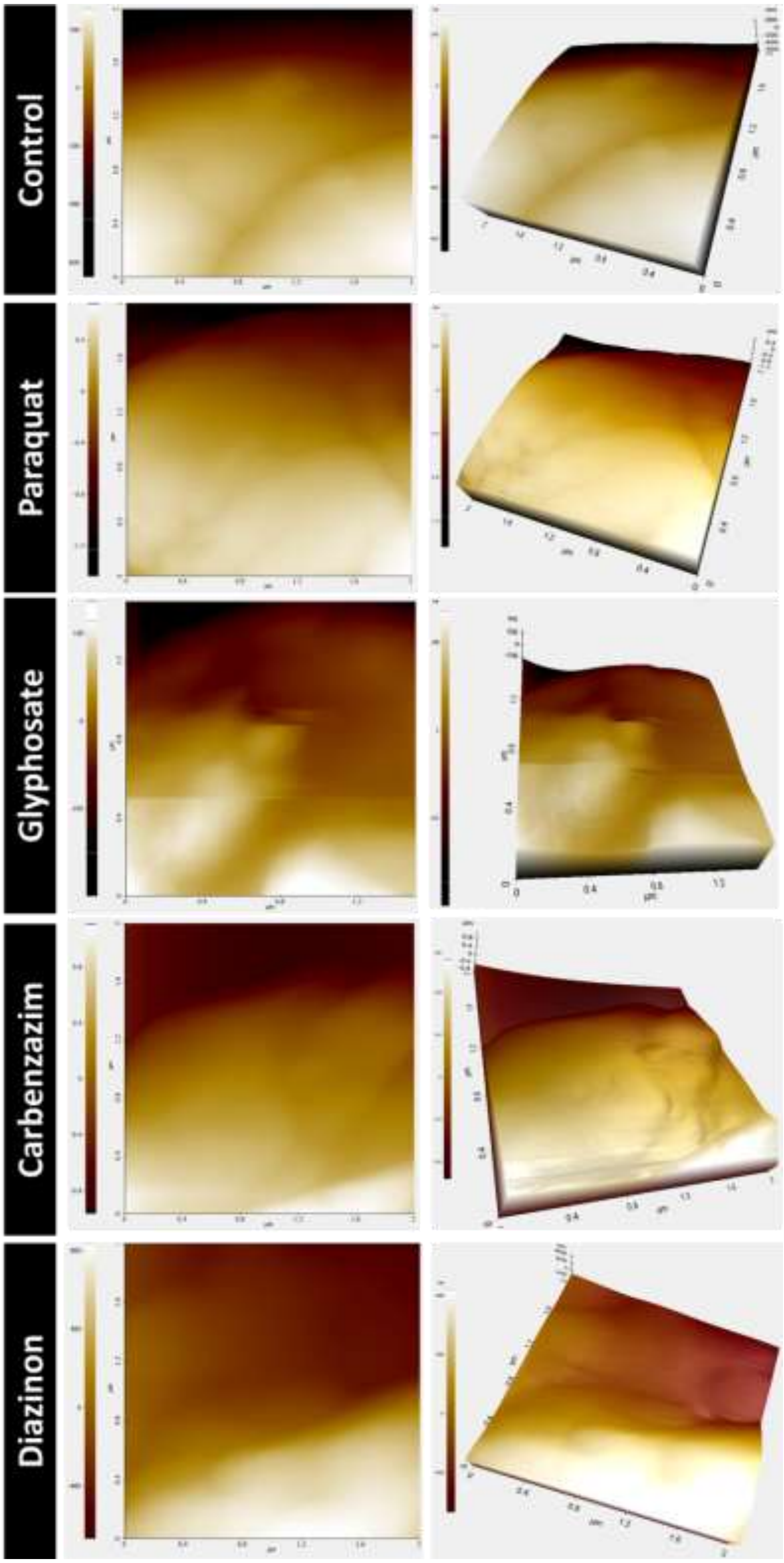

Figure S5. AFM Microscopy for Determining the Roughness of *C. sorokiniana*

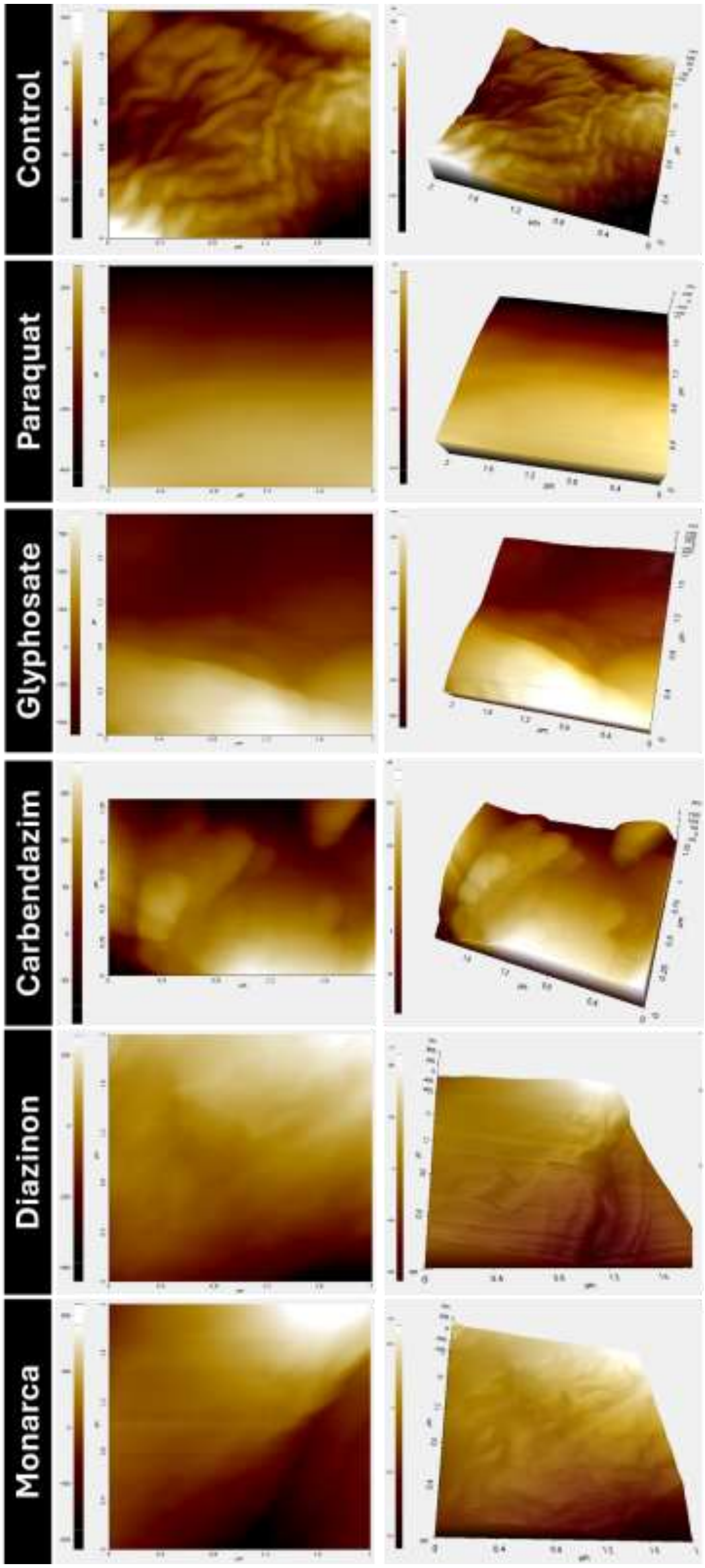

Figure S6. AFM Microscopy for Determining the Roughness of *Scendesmus* sp

Table S5. Analysis of roughness data

Sample	Paraquat	Glyphosate	Carbendazim	Diazinon	Monarca
C.sorokiniana	Significant increase in total roughness Possible surface damage or significant deposit	High roughness, although lower than Paraquat and Carbenzazim	High roughness. Significant effect, but smaller than Paraquat	Low roughness. More controlled surface damage	-
Scenedesmus sp	Elevated Sa, but low Sq. Possible generalized roughness without high peaks.	Maximum Sa, Sq, and Sz. Extremely altered surface. Possible severe damage or macroscopic accumulation	Very low Sz. Very smooth surface.	Higher Sz. Intense changes in relief. Obvious structural damage.	Moderate roughness. Clear but minor alteration
